# Combined serine protease PRSS22 and CEA mRNA analysis identifies the majority of colon cancer patients that recur within 12 years

**DOI:** 10.3389/fonc.2025.1628069

**Published:** 2025-08-20

**Authors:** Alaa Mohamed, Manar AbdelMageed, Faten Zahran, Nabila Zein, Lina Olsson, Gudrun Lindmark, Marie-Louise Hammarström, Sten Hammarström, Basel Sitohy

**Affiliations:** ^1^ Department of Clinical Microbiology, Umeå University, Umeå, Sweden; ^2^ Department of Diagnostics and Intervention, Umeå University, Umeå, Sweden; ^3^ Department of Biochemistry, Faculty of Science, Zagazig University, Zagazig, Egypt; ^4^ Department of Pathology and Clinical pathology, Faculty of Veterinary Medicine, Badr University in Cairo (BUC), Badr City, Egypt; ^5^ Department of Clinical Sciences, Lund University, Lund, Sweden

**Keywords:** colon cancer, serine proteases, PRSS3, PRSS22, CEA, regional lymph nodes, prognosis, mRNA analysis

## Abstract

**Introduction:**

Proteases play an important role in tumor progression. The predictive efficacy of proteases PRSS3 and PRSS22 mRNA levels for predicting relapse in surgically treated colon cancer (CC) patients was assessed.

**Methods:**

mRNA expression was quantified in 371 half lymph nodes (LNs) from 121 CC patients, 77 control LNs (13 patients), 66 primary colon tumors, and 30 normal colon tissues of these patients. Patients were also stratified according to their CEA mRNA level. The occurrence of relapse following curative surgery was evaluated using the Cox regression and Kaplan-Meier survival model analyses. Protein expression was examined through immunohistochemistry.

**Results:**

PRSS22 was superior to PRSS3 in identifying patients at risk of recurrence. Thus, high PRSS22 levels in LNs identified 76.5% of those who recurred, while PRSS3 only identified 17.6% of these patients and these were in TNM stages III and IV. The Kaplan-Meier analysis indicated that CC patients exhibiting elevated PRSS22 levels in lymph nodes experienced a reduction in survival time, averaging 37 months over the follow-up period (*p* = 0.009) and a 3-fold increased hazard risk (1.3–6.0; *p* = 0.01). In the group with low PRSS22 levels, only one patient experienced relapse at the 12-year follow-up when CEA mRNA analysis was included. A fraction of CEA-positive tumor cells expressed PRSS22 protein.

**Conclusion:**

The importance of the secreted serine protease, S1 family member PRSS22 in tumor progression is highlighted. It shows promise as a biomarker for CC prognosis and as a target to prevent tumor spread by inhibiting its enzymatic activity.

## 1 Introduction

Colorectal cancer (CRC) ranks as the second most common cause of cancer-related fatalities worldwide. Cancer death is strictly correlated with distant metastasis (1, 2). Surgery serves as the main treatment choice for CRC and is frequently paired with additional therapies, e.g., radiotherapy, chemotherapy, and anti-angiogenic therapy (3–5). Despite the higher rate of initial cure in recent years, 20-30% of CRC patients suffer from progressive metastasis and develop recurrence (6). One important reason for the high recurrence rate after curative surgery is the poor precision of the method used to detect malignant cells in the draining lymph nodes. The standard procedure is histopathology, in which only a small part of individual lymph nodes is used for analysis. Moreover, histopathology does not allow the classification of the tumor cells in the node into different risk groups depending on the properties of the tumor cells. Therefore, alternative methods for detection and risk group classification of malignant cells in the draining lymph nodes are urgently needed to guide oncologists in the choice of post-operative treatments.

mRNA biomarker analysis represents a highly promising alternative approach for assessing the risk of recurrence, enabling the assessment of the total lymph node volume and quantification of novel biomarkers exhibiting varying characteristics of the tumor cells (7). We previously studied mRNA biomarkers that correlated with poor prognosis in colon cancer (CC) patients, such as leucine-rich repeat-containing G protein-coupled receptors 5 and 6 (LGR5 and LGR6), as well as C-X-C motif chemokine ligands 16 and 17 (CXCL16 and CXCL17) (8–11). ColoNode, a novel prognostic tool, was found recently to exceed traditional histopathology in predicting colon cancer relapse and risk stratification by integrating mRNA expression analysis of five biomarkers [carcinoembryonic antigen cell adhesion molecule-5 (CEACAM5), kallikrein related peptidase-6 (KLK6), mucin-2 (MUC2), periostin (POSTN), solute carrier family 35 member D3 (SLC35D3)] in half of the lymph node volume (12).

KLK6 is a key biomarker in the ColoNode test. It is a serine protease belonging to the S1 MEROPS family of proteases (13). This family contains 118 members, many of which are secreted proteases acting in the extracellular environment. As excellently summarized in the recent review by Radisky, the activity of extracellular proteases is crucial in tumor proliferation and metastasis through various means, including remodeling of the extracellular matrix, cleavage of transmembrane proteins, activation of zymogens, degradation of inhibitors, etc (13). In this study, we have focused on another secreted protease, namely, protease, serine S1 family member 22 (PRSS22), also designated tryptase ε. It belongs to a different subfamily of proteases than KLK6 containing 7 expressed members (14). PRSS22 was detected in the normal adult esophagus and trachea at high levels and moderate levels in the thyroid, pancreas, placenta, and prostate, but not in the intestine including the colon as determined at the RNA level (14). Fetal lung expressed even higher levels than adult lung indicating that its expression is developmentally regulated (14). Most interestingly, PRSS22 is expressed and released by several types of cancer cell lines including colon adenocarcinomas (14, 15). Earlier studies have indicated that PRSS22 contributes to cancer cell development in human hepatocellular carcinoma (16), that overexpression of PRSS22 facilitated the breast cancer cells invasion and that suppression of PRSS22 was restrictive (17). In spite of all indications of an important role in tumor development and progression, PRSS22 has only been studied to a limited degree in CC. Therefor we chose this biomarker for investigation of its prognostic value in CC and its utility in identifying high-risk patients for post-operative treatment.

For comparative reasons, we have also analyzed the mRNA expression levels of PRSS3. PRSS3, also known as mesotrypsin, is a trypsin isoform that is a pancreatic-derived serine protease involved in intestinal digestion (18). There is an ongoing debate over PRSS3’s involvement in tumor growth; whereas some studies attribute a positive role (19–21), on the other hand, others claim that it has a tumor-suppressive role (22, 23).

High PRSS3 levels were associated with enhanced metastatic potential and predicted poor survival rates in non-small cell lung cancer and pancreatic cancer (20, 21). By contrast, many studies have revealed that in bladder, stomach, and esophagus cancers, promoter hypermethylation usually silences PRSS3 expression, pointing to a possible tumor suppressive function for PRSS3 (22, 23).

This study investigates the prognostic implications of PRSS22 and PRSS3 mRNA levels in CC patients’ lymph nodes (LNs) via qRT-PCR assay for absolute quantification. The 18S rRNA level is used to normalize the serine protease levels. We found that PRSS22 is an efficient marker for predicting the disease-free survival of CC patients who received curative surgery and that it complements mRNA analysis of CEA.

## 2 Materials and methods

### 2.1 Patient cohort and tissue specimens collection for mRNA expression analysis

One hundred and twenty-one patients in whom locally radical tumor resection for CC was carried out were included on a continuous basis at two Swedish sites, the Norrland University Hospital in Umeå and the Helsingborg Hospital in Helsingborg from November 2001 until February 2008. Inclusion criteria were primary surgery with intention to cure, willingness to participate in the study and no other cancer, except skin cancer excluding melanoma. LNs were collected from the resected specimen and bisected by the surgeon in the operating room. One half was formalin-fixed, embedded in an individual paraffin block and used in routine histopathology examination for pN classification. The other half was snap-frozen as fresh tissue and stored at -70°C until RNA extraction. A total of 371 LNs, on average 3 per patient (range 1-13), were collected. Of these, 70 LNs came from 23 patients in stage I, 186 LNs from 52 patients in stage II, 86 LNs from 37 patients in stage III, and 29 LNs from 9 patients in stage IV. Routine histopathological examination reported 20 LNs metastatic [H&E(+)] and 351 LNs non-metastatic [H&E(-)]. Fresh specimens of the primary tumor were gathered from 66 of the 121 CC patients. No patients received preoperative treatment. Tumor stage distribution was as follows: pT2 (n=13), pT3 (n=42), and pT4 (n=11). Normal colon specimens were gathered from the resection margins of tumors, distant from macroscopically detectable lesions, of 30 CC patients. Seventy-seven control LNs from 13 patients, eleven had ulcerative colitis, one had lipoma and one had Crohn’s disease. Clinicopathological characteristics of patients donating tissues for mRNA expression analysis are shown in **Table 1**.

**Table 1:** Clinical characteristics of colon cancer and control patients who donated primary tumor tissue, normal colon tissue and lymph nodes for mRNA and immunohistochemistry analyses.

Types of analyzed tissue	Type of analysis	n^#^	Gender		Age		TNM			
			Male	Female	Median	Range	I	II	III	IV
Primary colon cancer tumor	mRNA	66	30	36	74	42-88	14	30	17	5
	IHC**^&^**	10	5	5	72	60-84	1	3	4	2
Normal colon	mRNA	30	17	13	72	57-85	7	17	4	2
	IHC	9	4	5	70	41-83	1	3	2	3
Lymph nodes of colon cancer patients	mRNA*	121	55	66	74	42-89	23	52	37	9
	IHC**	10	2	8	80	71-91	1	2	7	0
Lymph nodes of control patients	mRNA	13	10	3	23	9-32	ǂ-	–	–	–

^#^n = number of patients who had donated tissue specimens of primary CC tumor tissue, normal colon tissue, and lymph nodes, respectively.

**^&^** IHC = Immunohistochemistry.

*Lymph nodes of colon cancer patients for mRNA analysis were retrieved from 121 colon cancer patients (n=371). Of these lymph nodes, 70 came from 23 patients in stage I, 186 from 52 patients in stage II, 86 from 37 patients in stage III, and 29 from 9 patients in stage IV.

** Lymph nodes of colon cancer patients for immunohistochemistry were collected from 10 patients (n=13); Seven lymph nodes were metastatic, i.e., H&E(+), from five patients in stage III. Six lymph nodes were non-metastatic, i.e., H&E(-). These nodes were from one patient in stage I, two patients in stage II, and two patients in stage III.

**^ǂ^-** = not applicable.

### 2.2 Patient cohort and tissue specimens collection for immunohistochemistry

Immunohistochemistry was performed on primary CC tumor samples from ten patients. Tumor stage distribution was as follows: pT2 (n=1), pT3 (n=6), and pT4 (n=3). Normal colon specimens were gathered from the resection margins of tumors, distant from macroscopically detectable lesions, of nine CC patients. LNs were from 10 patients (n=13). Seven LNs were metastatic, i.e., H&E(+), from five patients in stage III. Six LNs were non-metastatic, i.e., H&E(-). These nodes were from one patient in stage I, two patients in stage II, and two patients in stage III. Clinicopathological characteristics of patients donating tissues for immunohistochemistry are shown in **Table 1**.

### 2.3 Expression levels of mRNAs in colon cancer cell lines, monocytes, fibroblasts, and B-cell lines

Total RNA extracts from 5 human CC cell lines (Caco2, HCT8, HT29, T84, and LS174T), a monocyte cell line (U937), primary foreskin fibroblasts (FSU), and two B cell lines (KR4 and CNB6) were from earlier studies (24–26).

### 2.4 Quantitative reverse transcriptase–polymerase chain reaction assay

A real-time qRT-PCR assay was established for absolute quantification of PRSS3 and PRSS22 mRNAs, employing primers in different exons, exon-spanning probes, and specific RNA copy standards. The PRSS3 mRNA assay detects four transcript variants: NM_007343.4, NM_002771.4, NM_001197097.3, and NM_001197098.1. The primers and probe sequences for PRSS3 mRNA were forward primer 5’-GGCAACACTCTGAGCTTTGGT-3’, reverse primer 5’-CGGAGCATCCAGGCACTT-3’, and probe 5’-TGACTACCCAGACGAGC-3’. For PRSS22 mRNA (NM_022119.4), the primers and probe sequences were forward primer 5’-CGTGAGCATCCAGAAGAATGG-3’, reverse primer 5’-AGCAGCACAGAGAACAGGTATGG-3’ and probe 5’-CAAGGACAACCTGAACAA-3’. TaqMan probes were labeled with Flourescein Amidite (FAM) as the 5′ reporter dye and a non-fluorescent quencher-minor groove binder (NFQ-MGB) as the quencher dye at the 3′ end. The amplicon’s size was 60 and 126 for PRSS3 and PRSS22, respectively. The qRT-PCR conditions: 60°C (5 minutes) and 95°C (1 minute) then proceeded with 45 cycles of 95°C (15 seconds) and 60°C (1 minute). RNA oligonucleotide standards (Dharmacon, Lafayette, CO, USA) were designed to match the qRT-PCR amplicon sequences, facilitating absolute quantification. Every qRT-PCR run included serially diluted RNA copy standards (10^3^–10^8^ copies/μL). mRNA concentrations in unknown samples were calculated from the standard curve and expressed as mRNA copies/μL. 18S rRNA concentrations were determined from standard curves obtained by qRT-PCR analysis of serial dilutions of total-RNA extracted from human peripheral blood mononuclear cells run in parallel with unknown samples. One unit of 18S rRNA corresponded to 10 pg RNA (27). PRSS3 and PRSS22 mRNA levels were given as copies/18S rRNA unit. qRT-PCR assays for CEA, G protein-coupled receptor 35 V2/3 (GPR35 V2/3), CXCL17, CXCL16, LGR5, LGR6, and KLK6 mRNAs were described previously (8–11, 28–30).

### 2.5 Reagents for immunohistochemistry

Rabbit polyclonal anti-human PRSS3 antibody (IgG, Cat. No. PA5-103175, ThermoFisher, Waltham, MA, USA), mouse monoclonal anti-human PRSS22 antibody (mAb; IgG2a, Cat. No. MA5-24341, ThermoFisher), and mouse anti-carcinoembryonic antigen (CEA: II-7, IgG1) mAb (Dako, Glostrup, Denmark) were used. Anti-rabbit IgG and anti-mouse IgG ImmPRESS^®^ enhancement kits were used as secondary reagents (Vector Laboratories, Burlingame, CA, USA) and 3,3′-diaminobenzidine was the substrate that was utilized (Vector Laboratories).

### 2.6 Immunohistochemical staining procedure

Fresh tissue specimens were washed with cold phosphate-buffered saline (PBS), snap-frozen in isopentane, precooled in liquid nitrogen, and stored at −70°C. The frozen tissue was sliced into 4–6 µm-thick sections using a cryo-microtome (MICROM HM505E, ThermoFisher). As described previously (31, 32), the sections were treated with 4% paraformaldehyde for 15 minutes, then air-dried, rehydrated in PBS, and immersed in PBS containing 2 mM NaN_3_ and 0.03% H_2_O_2_ at 37°C for inhibition of endogenous peroxidase activity. Sections were treated with 0.2% bovine serum albumin in PBS, then incubated with 2.5% horse serum blocking solution (ImmPRESS,Vector Laboratories) at 25°C for blocking non-specific binding. Sections were immunostained using primary antibodies, followed by ImmPRESS anti-mouse/rabbit IgG. Peroxidase activity was visualized with 3,3′-diaminobenzidine (0.05%) in Tris buffer (pH 7.6), and methyl green served as a counterstain. Anti-CEA mAb was used as a positive control instead of the primary antibody.

### 2.7 Statistical analysis

The statistical significance of differences in mRNA levels between primary CC tumors and normal colon tissues was evaluated using the two-tailed Mann-Whitney rank sum test. The Kruskal-Wallis one-way analysis of variance (ANOVA) test, followed by Dunn’s multiple comparison *post hoc* test, was employed to analyze differences in mRNA levels in LNs from various TNM stages, as well as in H&E(+) versus H&E(-) and control LNs, and LNs with different CEA levels. Furthermore, the non-parametric Spearman correlation coefficient test was used to analyze the correlation between PRSS3 and PRSS22 mRNA levels as well as LGR6, LGR5, CXCL16, CXCL17, CEA, GPR35V2/3, and KLK6 mRNA levels. The statistical calculations were performed using GraphPad Prism 9 (GraphPad Software, San Diego, CA, USA). The SPSS software (IBM Corporation, Armonk, NY, USA) was used for statistical analyses of differences in disease-free survival time between patient groups, as well as tests of risk for disease recurrence, using univariate Cox regression and the Kaplan-Meier survival model combined with the log-rank test. A *p*-value of less than 0.05 was considered statistically significant.

## 3 Results

### 3.1 Differential mRNA expression of PRSS3 and PRSS22 in primary CC tumors versus normal colon tissue, CC cell lines, and immune cell lines and confirmation of expression at the protein level

**Figure 1:**
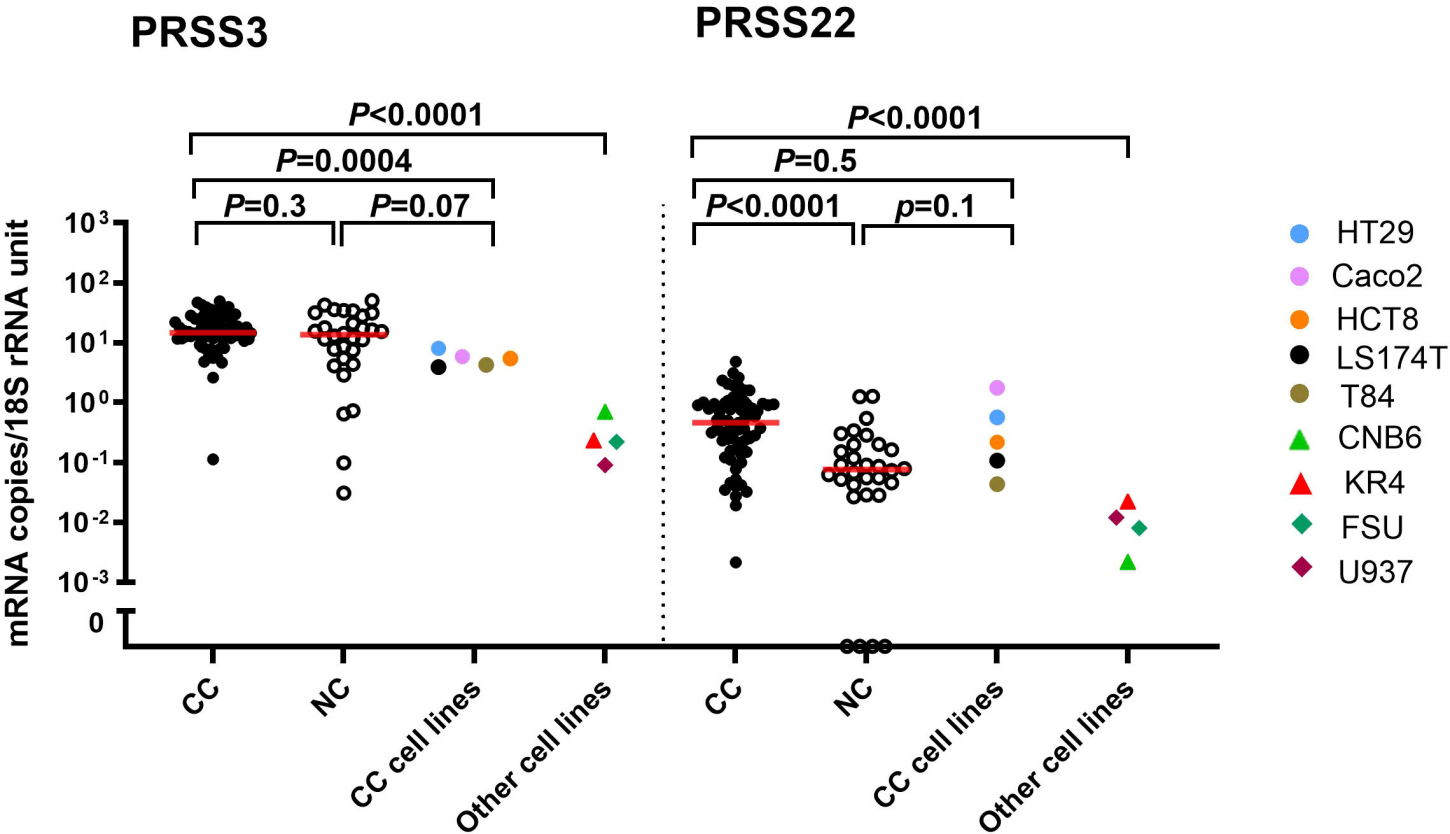
PRSS3 and PRSS22 mRNA expression levels in primary colon cancer (CC) tissue (n=66), normal colon (NC) tissue (n=30), five colon cancer cell lines (HT29, Caco2, HCT8, LS174T, T84), and in other cell lines; two B cell lines (CNB6, KR4), a monocyte cell line (U937), and primary foreskin fibroblast cells (FSU). Red horizontal lines indicate median values. Cell lines are color-coded as explained in the figure. *p*-values were calculated by a two-tailed Mann-Whitney test.

Primary CC tumors displayed a 6-fold increase in the median mRNA expression level of PRSS22 (0.44 versus 0.07 mRNA copies/18S rRNA unit, respectively) compared to normal colon tissues (*p* < 0.0001; **Figure 1**). The five colon carcinoma cell lines analyzed expressed PRSS22 mRNA at similar levels to the primary tumors with Caco2 cells expressing the highest levels and T84 the lowest level (**Figure 1**). Immune cells and fibroblasts expressed still lower levels, although the difference was not large.

In contrast, no statistically significant difference was observed in PRSS3 mRNA levels in primary tumor compared with normal colon tissue (14.5 and 13.6 mRNA copies/18S rRNA unit, respectively; *p* = 0.3). All five colon carcinoma cell lines expressed PRSS3 at the same level, slightly lower than the median level of primary tumors (**Figure 1**). The expression of PRSS3 was significantly higher than that of PRSS22 in CC cell lines and the fibroblast and immune cell lines (**Figure 1**). Comparative expression analysis of PRSS22 and PRSS3 in the primary tumors revealed that they were not significantly correlated (*p=*0.1; r=0.2).

**Figure 2:**
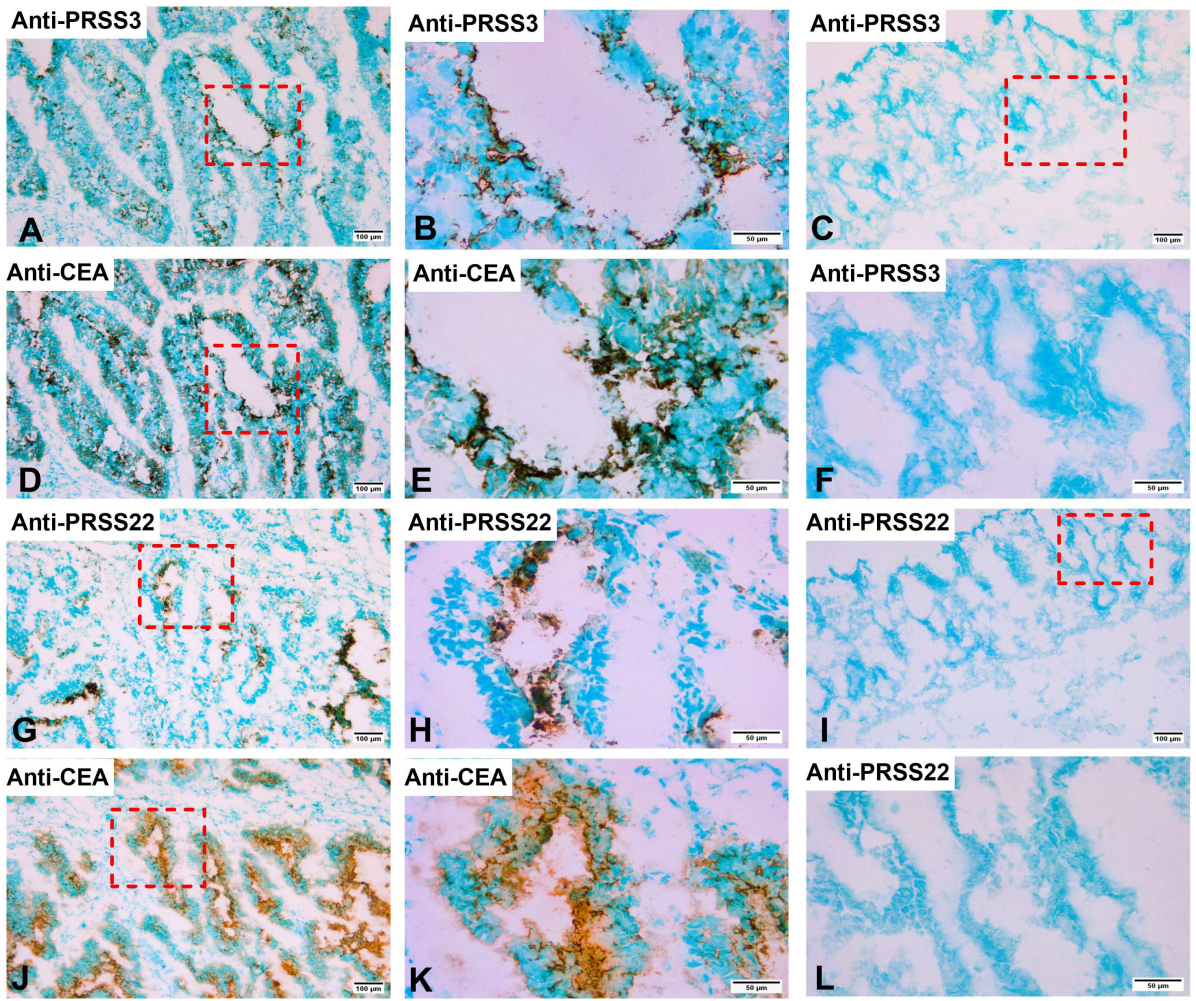
Immunoperoxidase stained tissue sections of a primary colon cancer tissue in **(A, B, D, E, G, H, J, K)** and normal colon tissue in **(C, F, I, L)**. **(A)**, consecutive section of **(D)** stained with rabbit anti-PRSS3 IgG, 100×. **(B)**, higher magnification of the indicated area in **(A)**, 400×. **(C)**, normal colon tissue stained with anti-PRSS3 IgG, 100×. **(D)**, primary colon cancer tissue stained with anti-CEA mAb, 100×. **(E)**, higher magnification of the indicated area in **(D)**, 400×. **(F)**, higher magnification of indicated area in **(C)**, 400×. **(G)**, consecutive section of **(J)** stained with anti-PRSS22 mAb, 100×. **(H)**, higher magnification of indicated area in **(G)**, 400×. **(I)**, normal colon tissue stained with anti-PRSS22 mAb, 100×. **(J)**, primary colon cancer tissue stained with anti-CEA mAb, 100×. **(K)**, higher magnification of indicated area in **(J)**, 400×. **(L)**, higher magnification of indicated area in **(I)**, 400×. The scale bar in **(A, C, D, G, I, J)**, that is figures with 100× original magnification, corresponds to 100 µm. The scale bar in **(B, E, F, H, K, L)**, that is figures with 400× original magnification, corresponds to 50 µm.

PRSS3 and PRSS22 protein expression in CC primary tumors and normal colon tissue was studied by the consecutive staining immunohistochemistry technique using anti-CEA mAb to identify tumor cells. Co-expression of CEA and PRSS3 was detected in a fraction of tumor cells (**Figures 2A, B, D, E**) or PRSS22 positive (**Figures 2G, H, J, K**), while such positive cells were not found in normal colon tissue (**Figures 2C, F, I, L**). It was noted that not all CEA-positive cells were PRSS22 positive.

### 3.2 Differential mRNA expression and protein confirmation of PRSS3 and PRSS22 in CC lymph nodes

**Figure 3:**
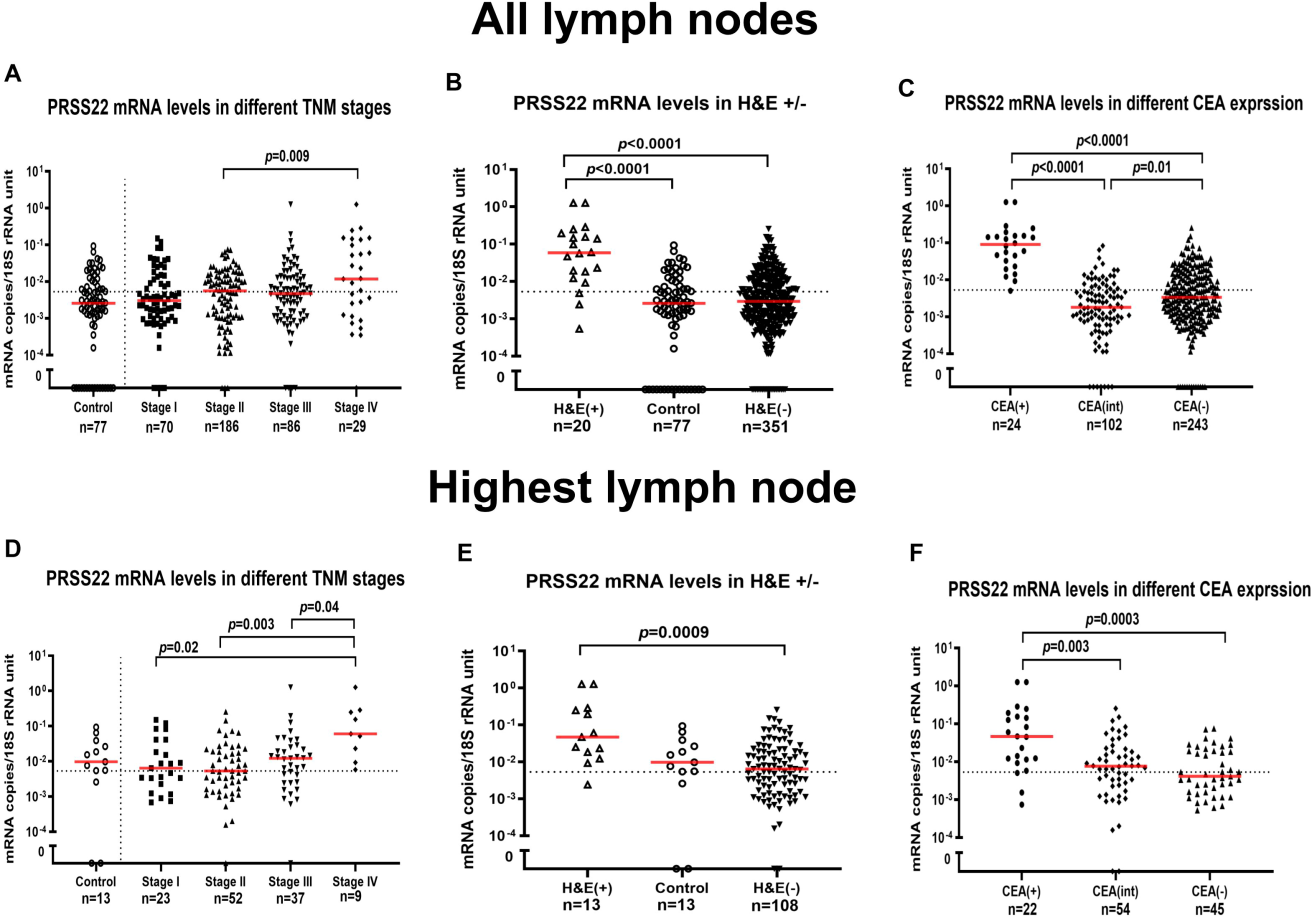
PRSS22 mRNA expression levels in all lymph nodes **(A–C)** and the lymph node with the highest level for each patient **(D–F)**. **(A, D)** show PRSS22 mRNA expression levels in lymph nodes from non-cancerous disease patients (Control) and colon cancer patients in different TNM-stages (Stage I–IV). **(B, E)** show PRSS22 mRNA expression levels in metastatic lymph nodes of colon cancer patients (H&E(+)), non-metastatic nodes of colon cancer patients (H&E(-)), and lymph nodes of non-cancerous disease patients (Control). **(C, F)** show PRSS22 mRNA expression levels in three groups categorized according to their CEA mRNA levels: CEA mRNA levels <0.013 copies/18S rRNA unit (CEA(-)), CEA mRNA levels between 0.013 and 3.67 copies/18S rRNA unit (CEA(int)), and CEA mRNA levels >3.67 copies/18S rRNA unit (CEA(+)). Dashed horizontal lines indicate the clinical cut-off value of 0.00533 mRNA copies/18S rRNA unit. n = number of analyzed lymph node samples. Red horizontal lines indicate median values. *p*-values were calculated by Kruskal–Wallis non-parametric ANOVA followed by *post hoc* Dunn’s test for multiple comparisons.

PRSS3 and PRSS22 mRNA levels were analyzed in 371 draining LNs from 121 CC patients (TNM stages I-IV) and 77 LNs from 13 non-cancerous controls. The median PRSS3 mRNA expression levels were similar in control LNs (0.01 copies/18S rRNA unit) and LNs in stages I-III (0.01, 0.008, and 0.01 mRNA copies/18S rRNA unit, respectively), but higher in stage IV (0.03 mRNA copies/18S rRNA unit). The difference between stage II and stage IV LNs was statistically significant (*p* = 0.008) (**Supplementary Figure S1A**). The median PRSS22 mRNA expression levels showed a trend of gradual increase across TNM stages: 0.003 (stage I), 0.006 (stage II), 0.005 (stage III), and 0.012 (stage IV) mRNA copies/18S rRNA unit. Control patients’ LNs showed low PRSS22 mRNA levels (0.003 mRNA copies/18S rRNA unit). A significant difference in PRSS22 levels was observed in stage IV versus stage II LNs (*p* = 0.009) (**Figure 3A**).

Routine histopathology detected metastasis in 20 LNs [H&E(+)], while the remaining 351 LNs were non-metastatic [H&E(-)]. Control LNs (n=77) are expected to be H&E(-). The PRSS3 median mRNA level was significantly elevated in H&E(+) LNs, exhibiting a 190-fold increase compared to H&E(-) LNs (1.9 and 0.01 mRNA copies/18S rRNA unit, respectively; *p* < 0.0001) (**Supplementary Figure S1B**). Similarly, the PRSS22 median mRNA level was significantly elevated in H&E(+) LNs, exhibiting a 20-fold increase compared to H&E(-) LNs (0.06 and 0.003 mRNA copies/18S rRNA unit, respectively; *p* < 0.0001). Control LNs had 20 times lower levels than H&E(+) LNs (*p* < 0.0001) (**Figure 3B**).

Finally, PRSS3 and PRSS22 mRNA expression levels were matched with CEA mRNA expression values using earlier reported values for this clinical material (28). LNs were divided into three groups: CEA(+) (>3.67 copies/18S rRNA unit), CEA(int) (0.013-3.67 copies/18S rRNA unit), and CEA(-) (<0.013copies/18S rRNA unit). The median PRSS3 mRNA expression values in the CEA(+), CEA(int), and CEA(-) groups were 2.3, 0.01 and 0.009 mRNA copies/18S rRNA unit, respectively (**Supplementary Figure S1C**). The median PRSS3 mRNA level was significantly higher in LNs of the CEA(+) group compared to LNs of the CEA(int) and the CEA(-) groups (*p* < 0.0001). The median PRSS22 mRNA levels in the CEA(+), CEA(int), and CEA(-) groups were 0.09, 0.002, and 0.003 mRNA copies/18S rRNA unit, respectively. PRSS22 mRNA expression levels were significantly higher in the CEA(+) group compared to the CEA(int) and CEA(-) groups (*p* < 0.0001) (**Figure 3C**).

To compare mRNA expression data of PRSS3 and PRSS22 with survival data from Kaplan-Meier and Cox regression analysis, we chose the LN with the highest level of PRSS3 and PRSS22 mRNA, respectively, to represent the patient. These results are shown in **Supplementary Figures S1D–F**, **Figures 3D–F**. For the PRSS3 marker, the result for the highest LNs was closely similar to those obtained for the entire LN material. Regarding the highest LNs of PRSS22, the levels were significantly higher in LNs of stage IV patients compared to LNs of patients in stage I, II, and III (**Figure 3D**). The significance of a higher median PRSS22 level in H&E(+) LNs compared to H&E(-) LNs remained (**Figure 3E**). Also, CEA(+) LNs had a significantly higher median level compared both CEA(int) and CEA(-) LN groups (**Figure 3F**).

**Figure 4:**
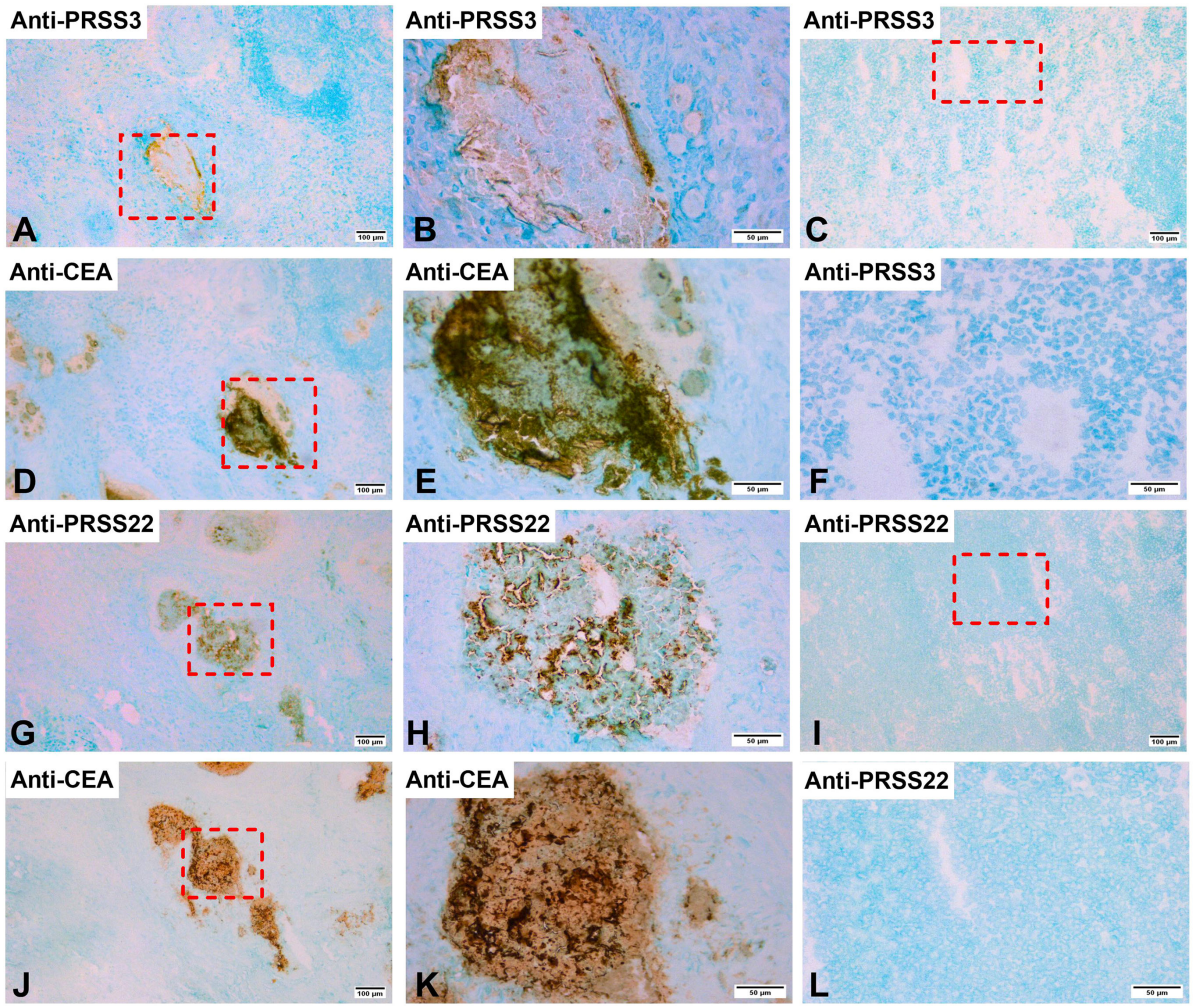
Immunoperoxidase stained tissue sections of lymph nodes of a colon cancer patient. **(A, B, D, E, G, H, J, K)** show sections of a metastatic lymph node [H&E(+)] and **(C, F, I, L)** a non-metastatic lymph node [H&E(-)]. **(A)** H&E(+) lymph node stained with anti-PRSS3 mAb in a consecutive section of **(D)**, original magnification 100×. **(B)** higher magnification of the area indicated by a hatched box in **(A)**, 400×. **(C)** H&E(-) lymph node stained with anti-PRSS3 mAb, original magnification 100x. **(D)** H&E(+) lymph node stained with anti-CEA mAb, original magnification 100×.**(E)** higher magnification of the area indicated by a hatched box in **(D)**, 400×. **(F)** higher magnification of the area indicated by a hatched box in **(C)**, 400×. **(G)** H&E(+) lymph node stained with anti-PRSS22 mAb in a consecutive section of **(J)**, original magnification, 100×. **(H)** higher magnification of the area indicated by a hatched box in **(G)**, 400x. **(I)** H&E(-) lymph node stained with anti-PRSS22 mAb, original magnification 100x. **(J)** H&E(+) lymph node stained with anti-CEA mAb, original magnification 100×.**(K)** higher magnification of the area indicated by a hatched box in **(J)**, 400×. **(L)** higher magnification of the area indicated by a hatched box in **(I)**, 400×. The scale bar in **(A, C, D, G, I, J)**, that is figures with 100× original magnification, corresponds to 100 µm. The scale bar in **(B, E, F, H, K, L)**, that is figures with 400× original magnification, corresponds to 50 µm.

Immunohistochemical analysis with anti-PRSS3, anti-PRSS22, and anti-CEA antibodies applied to H&E(+) LNs demonstrated that a fraction of the tumor cells in the LNs, displayed positive staining for both PRSS3 (**Figures 4A, B, D, E**) and PRSS22 (**Figures 4G, H, J, K**). CC patients’ H&E(-) LNs did not exhibit any positive staining with anti-PRSS3 and anti-PRSS22 (**Figures 4C, F, I, L**). PRSS22 appears to be more strongly expressed compared to PRSS3.

### 3.3 Correlation of PRSS3, PRSS22, LGR6, LGR5, CXCL16, CXCL17, CEA, GPR35V2/3 and KLK6 mRNA expression levels in CC lymph nodes

The mRNA expression levels of LGR6, LGR5, CXCL16, CXCL17, CEA, GPR35V2/3, and KLK6 have previously been determined in the LNs studied in this work (8–11, 28–30). Correlation coefficients (r) and significance levels (*p*) for mRNAs biomarker with PRSS3 and PRSS22 in overall CC patients and stratified by TNM stage are shown in **Table 2**. Of particular interest are the results from stage III and IV patients where a large fraction of the cells in the LNs are expected to be tumor cells. Both PRSS3 and PRSS22 showed a strong highly significant positive correlation with all seven biomarkers with r-values ranging from 0.2-0.5 for LNs of stage III and 0.5-0.9 for Stage IV patients indicating that these biomarkers are probably expressed by the same population of tumor cells. There was also a strong correlation between PRSS3 and PRSS22 in all four TNM stages.

**Table 2:** Correlations between levels of PRSS3 and PRSS22 mRNAs and CEA, CXCL16, CXCL17, GPR35 V2/3, LGR5, LGR6 and KLK6 mRNA levels in lymph nodes of colon cancer patients.

LN group		CEA		CXCL17		CXCL16		GPR35 V2/3		LGR5		LGR6		KLK6		PRSS3	
		r	*p*-value	r	*p*-value	r	*p*-value	r	*p*-value	r	*p*-value	r	*p*-value	r	*p*-value	r	*p*-value
PRSS3	All CC° LNs*	0.3	<0.0001	0.2	0.0004	0.2	<0.0001	0.2	0.002	0.2	0.0001	0.4	<0.0001	0.3	<0.0001		
	TNM Stage I LNs	0.1	0.4	0.2	0.2	0.2	0.2	0.1	0.4	0.2	0.2	0.3	0.01	-0.2	0.06		
	TNM Stage II LNs	0.04	0.6	-0.03	0.7	0.1	0.08	-0.01	0.9	0.01	0.9	0.3	<0.0001	0.04	0.5		
	TNM Stage III LNs	0.5	<0.0001	0.2	0.04	0.2	0.03	0.2	0.05	0.3	0.001	0.5	<0.0001	0.4	<0.0001		
	TNM Stage IV LNs	0.8	<0.0001	0.8	<0.0001	0.6	0.0003	0.8	<0.0001	0.6	0.0002	0.9	<0.0001	0.8	<0.0001		
PRSS22	All CC LNs	0.03	0.5	0.2	0.001	0.3	<0.0001	0.2	0.0002	0.15	0.004	0.5	<0.0001	0.21	<0.0001	0.4	<0.0001
	TNM Stage I LNs	-0.2	0.09	0.04	0.8	0.01	0.9	-0.1	0.7	-0.01	0.9	0.3	0.02	-0.12	0.3	0.4	0.0004
	TNM Stage II LNs	-0.2	0.01	0.07	0.3	0.2	0.01	0.07	0.3	-0.03	0.7	0.4	<0.0001	-0.12	0.09	0.3	<0.0001
	TNM Stage III LNs	0.3	0.01	0.2	0.06	0.3	0.001	0.4	0.001	0.4	<0.0001	0.5	<0.0001	0.4	0.0006	0.4	<0.0001
	TNM Stage IV LNs	0.7	<0.0001	0.6	0.001	0.6	0.0002	0.8	<0.0001	0.6	0.001	0.8	<0.0001	0.8	<0.0001	0.8	<0.0001

The correlation coefficients (r) and the *p*-values were calculated by two-tailed Spearman’s rank order correlation test.

### 3.4 Correlation of PRSS3 and PRSS22 mRNA expression levels in the primary tumor and highest CC lymph nodes

**Table 3** summarizes the result of a correlation analysis between mRNA expression levels of PRSS3 and PRSS22 in the primary CC tumor versus LNs, using the LN expressing the highest level in the comparison. There was a marked difference between the two proteases, while PRSS22 showed a high degree of correlation in pairwise comparison between LNs and primary tumors when all LNs, stage II and stage III LNs were compared, only stage III LNs showed a significant correlation for PRSS3. Stage IV patients were too few (n=5) to allow an interpretation.

**Table 3:** Pairwise comparisons of levels in the primary tumor and the highest lymph node of PRSS3 and PRSS22 mRNAs in colon cancer patients.

Patient group	PRSS3		PRSS22	
	r	*p*-value	r	*p*-value
All CC patients (n=66)	0.1	0.2	0.3	0.008
Stage I patients (n=14)	-0.04	0.9	0.3	0.4
Stage II patients (n=30)	-0.003	0.9	0.4	0.04
Stage III patients (n=17)	0.6	0.01	0.5	0.05

The correlation coefficients (r) and the *p*-values were calculated by two-tailed Spearman’s rank order correlation test.

### 3.5 Clinical consequence of PRSS3 and PRSS22 mRNA expression levels in CC lymph nodes - prediction of recurrence

The association between PRSS3 and PRSS22 mRNA levels in LNs and disease recurrence and disease-free survival time after surgery was studied. We employed Cox regression and Kaplan-Meier analysis followed by the log-rank test to evaluate the biomarker predictive value and survival outcomes. For each CC patient, the LN with the highest mRNA level was selected, and cut-off values for high/low recurrence risk were established for each biomarker. These survival analyses are summarized in **Supplementary Table S1**; **Table 4**.

**Table 4:** Comparative analysis of average survival time after surgery and risk for recurrence of disease of colon cancer patients with PRSS22(-) and PRSS22(+) lymph nodes and combination with CEA.

Patient group	Category ^a^	Number of patients in each group stratified by TNM stage				Total	5-year follow-up after surgery					12- year follow-up after surgery				
		Stage I	Stage II	Stage III	Stage IV		Disease-free survival ^b^			Risk for recurrence^c^		Disease-free survival			Risk for recurrence	
							Average (Months)	Difference (Months)	*p*-value	Hazard ratio (95%CI)	*p*-value	Average (Months)	Difference (Months)	*p*-value	Hazard ratio (95%CI)	*p*-value
All CC patients	PRSS22(-)	11	26	13	0	50	55	7	0.007	3.0	0.01	127	37	0.009	3.0	0.01
	PRSS22(+)	12	26	24	9	71	48			(1.3-7.0)		90			(1.3-6.0)	
CEA(int) plus CEA(+) CC patients ^d^	PRSS22(-)	5	15	5	0	25	59	14	<0.001	15.0	0.008	143	61	<0.001	14.0	0.009
	PRSS22(+)	8	15	19	9	51	45			(2.0-111.0)		82			(2.0-106.0)	
CEA(int) CC patients ^e^	PRSS22(-)	4	15	3	0	22	59	9	0.02	8.3	0.04	143	46	0.02	8.3	0.04
	PRSS22(+)	7	14	10	1	32	50			(1.1-64.4)		97			(1.1-65.0)	
CEA(-) CC patients ^f^	PRSS22(-)	6	11	8	0	25	51	6	0.4	0.6	0.4	108	1	0.8	0.8	0.8
	PRSS22(+)	4	11	5	0	20	57			(0.1-2.3)		107			(0.3-2.6)	

a Colon cancer patients divided into two groups PRSS22(-) and PRSS22(+) according to the median of mRNA expression in the highest lymph nodes in the colon cancer patients in TNM stage II (0.00533 mRNA copies/18S rRNA unit).

b Mean survival time after surgery calculated by cumulative survival analysis according to Kaplan-Meier analysis.

c Hazard ratio for recurrence with 95% confidence interval (CI) as calculated according to univariate COX regression analysis.

d CEA(int) plus CEA(+) group: colon cancer patients with CEA mRNA levels >0.013 copies/18S rRNA unit.

e CEA(int): colon cancer patients with CEA mRNA levels between 0.013 and 3.67 copies/18S rRNA unit.

f CEA(-): group colon cancer patients with CEA mRNA levels ≤0.013 copies/18S rRNA unit.

The result of survival analysis with PRSS3 is given in the **Supplementary Material**. Briefly, the best division between PRSS3(+) and PRSS3(-) was determined according to the median value of PRSS3 mRNA expression in LNs from CC patients in the CEA (+) group (2.3 mRNA copies/18S rRNA unit), associated with a 16-month survival difference (*p* = 0.008) at 5 years. A drawback with PRSS3 as a prognostic marker is that the negative group contained a large number of patients who died of CC (**Supplementary Table S1**; **Supplementary Figure S2**). Almost all these patients were TNM stage III and IV patients.

**Figure 5:**
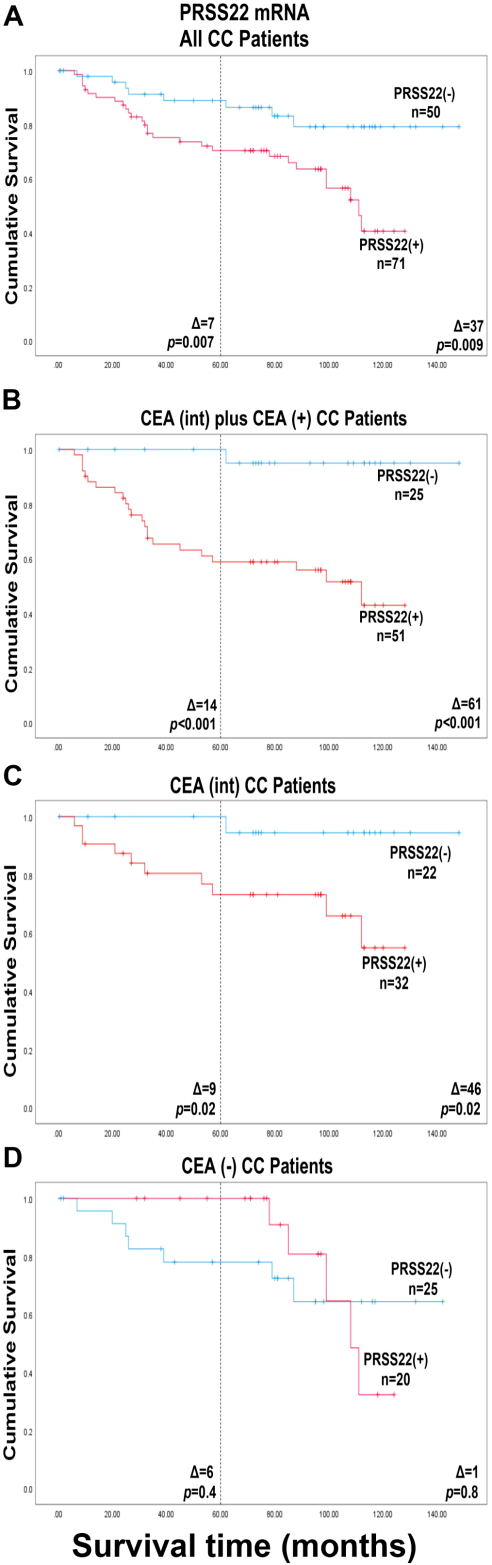
Kaplan-Meier cumulative survival curves for CC patients divided into two groups, PRSS22(-) and PRSS22(+), according to the median of the expression level in the highest lymph nodes of the CC patients in stage II (0.00533 mRNA copies/18S rRNA unit). **(A)**, all 121 colon cancer patients in the study represented by their highest lymph node. **(B)**, patients with CEA mRNA lymph node levels >0.013 mRNA copies/18S rRNA unit (CEA(int) plus CEA(+) CC patients; n = 76). **(C)**, patients with CEA mRNA levels between 0.013 and 3.67 copies/18S rRNA unit (CEA(int) CC patients; n = 54). **(D)**, patients with CEA mRNA levels <0.013 mRNA copies/18S rRNA unit (CEA(-) CC patients; n = 45). The patients were followed for 12 years. The dashed line indicates a 5-year follow-up after surgery. Differences in disease-free survival time after surgery between the two groups are given as a Δ-value in months. *p*-values were calculated using log-rank test of survival analysis. n = number of patients in the respective group.

In contrast, fewer patients (8 patients) had recurred in the PRSS22(-) group compared to the PRSS22(+) group (26 patients) (**Figure 5A**). The cut-off level of PRSS22 was 0.00533 mRNA copies/18S rRNA unit, corresponding to the median mRNA expression level in the highest LNs of CC patients in TNM stage II. The group with high PRSS22 mRNA levels (PRSS22(+), n = 71) had a 3.0-fold higher recurrence rate than the group with low PRSS22 mRNA levels in their highest LN (PRSS22(-), n = 50) at 5 and 12 years follow up (*p* = 0.01 at both timepoints). After surgery, differences in mean disease-free survival time were 7 months (5 years follow-up, *p* = 0.007) and 37 months (12 years follow-up, *p* = 0.009).

Combining PRSS22 mRNA analysis with CEA mRNA analysis improved the distinction between patients who died of CC and those who survived (**Figures 5B, C**; **Table 4**). The combined CEA(+) plus CEA(int) group (CEA mRNA levels > 0.013 mRNA copies/18S rRNA unit) (28), included 63% of the patients. The PRSS22(+)group (n=51) showed a 15-fold increased recurrence rate versus the PRSS22(-)group (n=25) at 5-year follow-up and a 14.0-fold increase at 12-year follow-up (*p* = 0.008, *p* = 0.009, respectively). The difference in mean disease-free survival time after surgery was 14 and 61 months, respectively (*p* < 0.001 at both timepoints).

A group of 45 patients had no CEA-positive LNs, suggesting presence of only very low numbers of tumor cells. Still, twelve patients in this group had recurred at 12-year follow-up (**Figure 5D**; **Table 4**). No statistically significant difference in outcomes was observed between PRSS22(+)CEA(-) and PRSS22(-)CEA(-) groups. At 5-year follow-up there was five of these twelve patients who were missed by the combined CEA and PRSS22 analysis and had recurred in CC. They constituted 4% of all the patients and 21% of the patients who had recurred 5 years after surgery.

### 3.6 Expression levels of PRSS3 and PRSS22 mRNAs in primary tumor did not allow division of patients into groups with different risk of recurrence

No significant difference in recurrence risk or disease-free survival time was observed in CC patients using either of the two serine protease PRSS3 and PRSS22 mRNAs with the level at the 25^th^, 50^th^ or 75^th^ percentile of primary CC tumors as cut-offs for division into two groups. Cut-off values were 11.6, 14.5, and 24.9 mRNA copies/18S rRNA unit for PRSS3 and 0.17, 0.44, and 0.93 mRNA copies/18S rRNA unit for PRSS22 (data not shown).

## 4 Discussion

This study pioneers the investigation of PRSS22 mRNA expression in regional LNs as a prognostic biomarker in colorectal cancer patients undergoing curative surgery for cure. A well-defined clinical material consisting of 121 CC patients followed for twelve years after surgery with LNs from patients of all four TNM stages was investigated. This material has been used to study the utility of 13 other biomarker mRNAs coding for different functional groups of proteins (8–11, 24, 26, 29, 33). For comparison, we also analyzed mRNA for another serine protease, namely PRSS3.

The key finding is that PRSS22 is a very strong biomarker that allows division of the CC patients into two subgroups - one with excellent survival, the PRSS22(-) group, and one with relatively poor survival, the PRSS22(+) group. Notably the PRSS22(-) group was large (50 patients = 41%). The second important finding was that PRSS22 mRNA analysis complements CEA mRNA analysis. Thus, by combining the result of analysis of the two biomarkers - one detecting tumor cells in LNs derived from the large intestine (*i.e.*, CEA) and one detecting a secreted protease (*i.e*., PRSS22), 64%, (22/34) of the patients that died of CC during the 12 years after operation were found in the marker positive group. In the combined marker negative group, only 1 patient (3%, 1/34) recurred during the 12-year observation period. Indeed, none of the patients in the minus group had recurrence within 5 years. The latter finding is so far unique for PRSS22. For example have the stem cell markers LGR4, LGR5 and LGR6 used on their own, similar or slightly better capacity to identify patients at risk for recurrence than PRSS22 (8, 9). However, in contrast to PRSS22 combined with CEA, these biomarkers combined with CEA failed to identify the potentially cured patients (8, 9), that is the PRSS22(-) patients in the CEA(+) plus CEA(int) group (**Figure 5B**).

Why did we miss 12 CC patients (10%) who recurred in this study? There are at least three possible reasonable explanations: 1) the number of LNs/patient was too small. In this material, only 2.3 LNs/patient were available for analysis. According to guidelines for histopathology analysis, 12 LNs/patient should be examined. 2) these patients represent a specific group that develops colon tumors by a pathway not involving PRSS22 or CEA. We did not find that the 12 patients who died of CC were unusual in terms of sex, age, TNM stage, or T stage. However, the result of the analysis of several of the genetic markers relevant to CRC were not available. 3) the cut-off level between positive and negative values was not precise enough due to the limited size of the clinical material. Further studies using a larger clinical material will answer the last question. A question that presents itself is if analysis of one or more additional markers could improve the results still further. It is interesting to note that immature colonocytes express low or very low levels of CEA (34) but the stem cell markers LGR4, LGR5 and LGR6 detect immature colonocytes (8, 9). Thus, including any of these markers in a trippel assay may improve the results further.

Our results with PRSS22 in CC patients are in line with those of Song et al. (17) on breast cancer, who reported that PRSS22 was overexpressed in patients with LN metastasis. Also, an earlier study reported that Hepsin, a type II transmembrane serine protease, plays a role in advanced-stage gastric cancer, exhibiting significant expression differences in patients with progressed disease (35). A very recent study by Xu and coworkers, using single-cell transcriptomics in primary tumors, reported PRSS22 as a hub gene in CRC (36). Of particular interest was that PRSS22 was shown to be linked to dysregulation of the tumor immune microenvironment, which might explain the importance of high expression of PRSS22 in LNs.

In contrast, PRSS3 had only limited prognostic value. It could however be used to categorize stage IV patients into two groups with significantly different risk for recurrence. Future studies will reveal whether this knowledge has clinical relevance.

What is the mechanism behind the important role that PRSS22 seems to play in tumor progression in CC? In breast cancer, PRSS22 is essential for cancer development and progression via triggering of the extracellular signal-regulated kinase 1/2 (ERK1/2) signaling pathway (17). Similarly, PRSS22 appears to influence tumor cell migration and invasion in hepatocellular carcinoma (16). Likely, one or more cellular processes are dysregulated by overexpression of this serine protease. Specific studies are needed to identify which processes are dysregulated by PRSS22 in CC.

KLK6, PRSS22, and PRSS3 are all secreted serine proteases, the first two identify CC tumor cells that are useful as tumor markers because they identify aggressive tumor cells. In contrast, PRSS3 has little use as a tumor marker in CC. One obvious explanation for the difference is that PRSS3 is also expressed in immune cells to a greater degree compared to the other two markers. Less obvious but perhaps also of importance is that the substrate specificity of the serine proteases is likely to be different from each other. This will have consequences in the tumor microenvironment both on which molecules are activated, and which are inhibited in their functions.

## 5 Conclusions

The importance of the secreted serine protease, S1 family member PRSS22, in tumor progression is highlighted. It shows promise as a prognostic biomarker for CC patients and as a target to prevent tumor spread by inhibiting its enzymatic activity.

## Data Availability

The original contributions presented in the study are included in the article/**Supplementary Material**. Further inquiries can be directed to the corresponding author.

## Supplementary material

Supplementary Figure 1 PRSS3 mRNA expression levels in all lymph nodes **(A–C)** and the lymph node with the highest level for each patient **(D–F)**. **(A, D)** show PRSS3 mRNA expression levels in lymph nodes from non-cancerous disease patients (Control) and colon cancer patients in different TNM-stages (Stage I–IV). **(B, E)** show PRSS3 mRNA expression levels in metastatic lymph nodes of colon cancer patients (H&E(+)), non-metastatic nodes of colon cancer patients (H&E(-)), and lymph nodes of non-cancerous disease patients (Control). **(C, F)** show PRSS3 mRNA expression levels in three groups categorized according to their CEA mRNA levels: CEA mRNA levels <0.013 copies/18S rRNA unit (CEA(-)), CEA mRNA levels between 0.013 and 3.67 copies/18S rRNA unit (CEA(int)), and CEA mRNA levels >3.67 copies/18S rRNA unit (CEA(+)). Dashed horizontal lines indicate the clinical cut-off value of 2.3 mRNA copies/18S rRNA unit. n = number of analyzed lymph node samples. Red horizontal lines indicate median values. *p*-values were calculated by Kruskal–Wallis non-parametric ANOVA followed by *post hoc* Dunn’s test for multiple comparisons.

Supplementary Figure 2 Kaplan-Meier cumulative survival curves for CC patients divided into two groups, PRSS3(-) and PRSS3(+), according to the median value of PRSS3 mRNA expression in lymph nodes from CC patients in the CEA(+) group (2.3 mRNA copies/18S rRNA unit). **(A)**, All 121 colon cancer patients in the study were represented by their highest lymph node. **(B)**, patients with CXCL16 mRNA levels >11.4 mRNA copies/18S rRNA unit (CXCL16(+) CC patients; n = 23). **(C)**, patients with LGR6 mRNA levels >0.0471 mRNA copies/18S rRNA unit (LGR6(+) CC patients; n = 30). The patients were followed for 12 years. The dashed line indicates a 5-year follow-up after surgery. Differences in disease-free survival time after surgery between the two groups are given as a Δ-value in months. *p*-values were calculated using log-rank test of survival analysis. n = number of patients in the respective group.
