# Supplementary figures and images for: Combined serine protease PRSS22 and CEA mRNA analysis identifies the majority of colon cancer patients that recur within 12 years
Source: Front Oncol. 2025 Aug 20;15:1628069. doi: 10.3389/fonc.2025.1628069 (PMC12406016; doi:10.3389/fonc.2025.1628069)

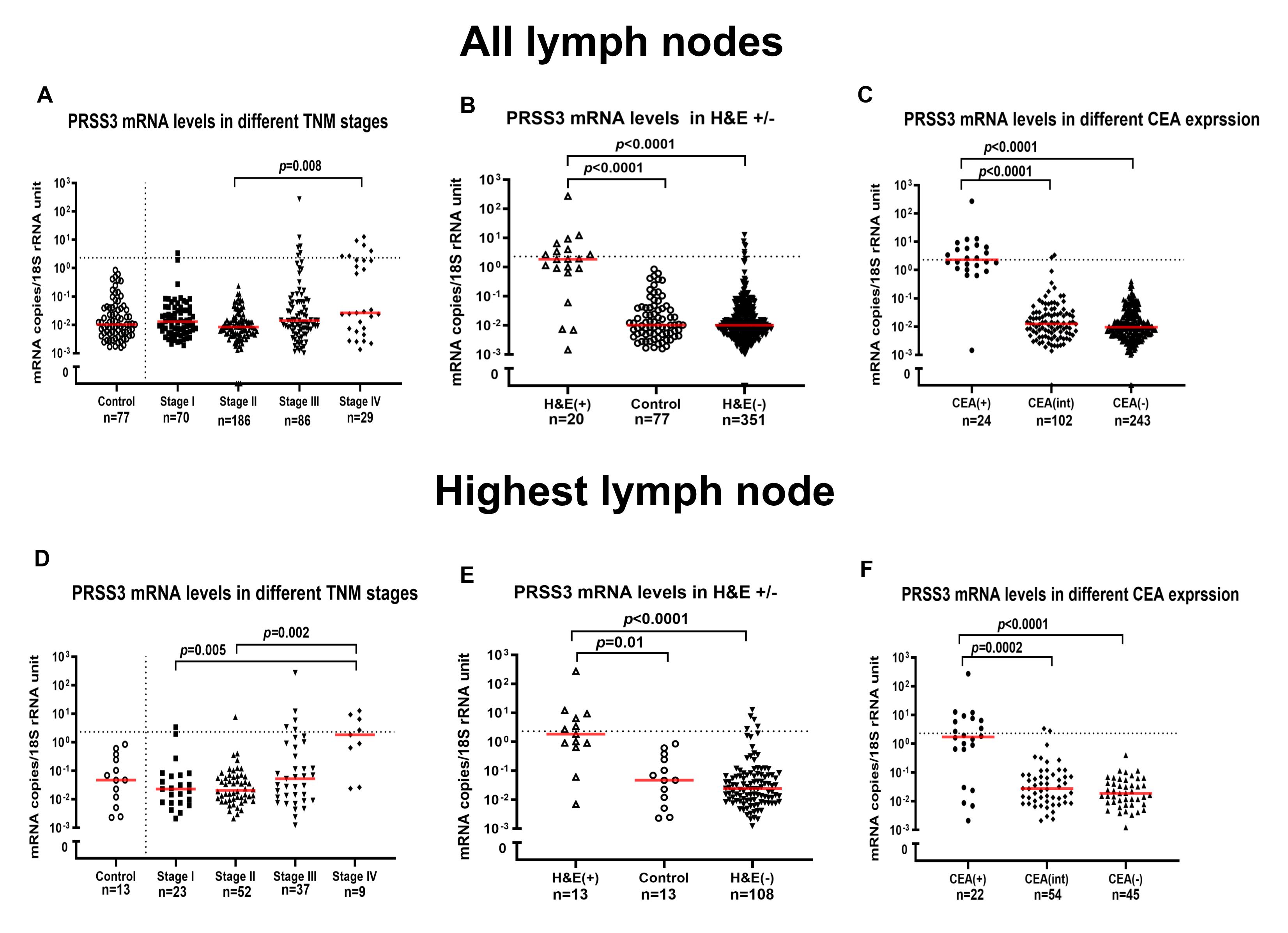

Supplement: Supplementary Figure 1 — PRSS3 mRNA expression levels in all lymph nodes (A–C) and the lymph node with the highest level for each patient (D–F). (A, D) show PRSS3 mRNA expression levels in lymph nodes from non-cancerous disease patients (Control) and colon cancer patients in different TNM-stages (Stage I–IV). (B, E) show PRSS3 mRNA expression levels in metastatic lymph nodes of colon cancer patients (H&E(+)), non-metastatic nodes of colon cancer patients (H&E(-)), and lymph nodes of non-cancerous disease patients (Control). (C, F) show PRSS3 mRNA expression levels in three groups categorized according to their CEA mRNA levels: CEA mRNA levels <0.013 copies/18S rRNA unit (CEA(-)), CEA mRNA levels between 0.013 and 3.67 copies/18S rRNA unit (CEA(int)), and CEA mRNA levels >3.67 copies/18S rRNA unit (CEA(+)). Dashed horizontal lines indicate the clinical cut-off value of 2.3 mRNA copies/18S rRNA unit. n = number of analyzed lymph node samples. Red horizontal lines indicate median values. p-values were calculated by Kruskal–Wallis non-parametric ANOVA followed by post hoc Dunn’s test for multiple comparisons. [file Image1.jpeg]

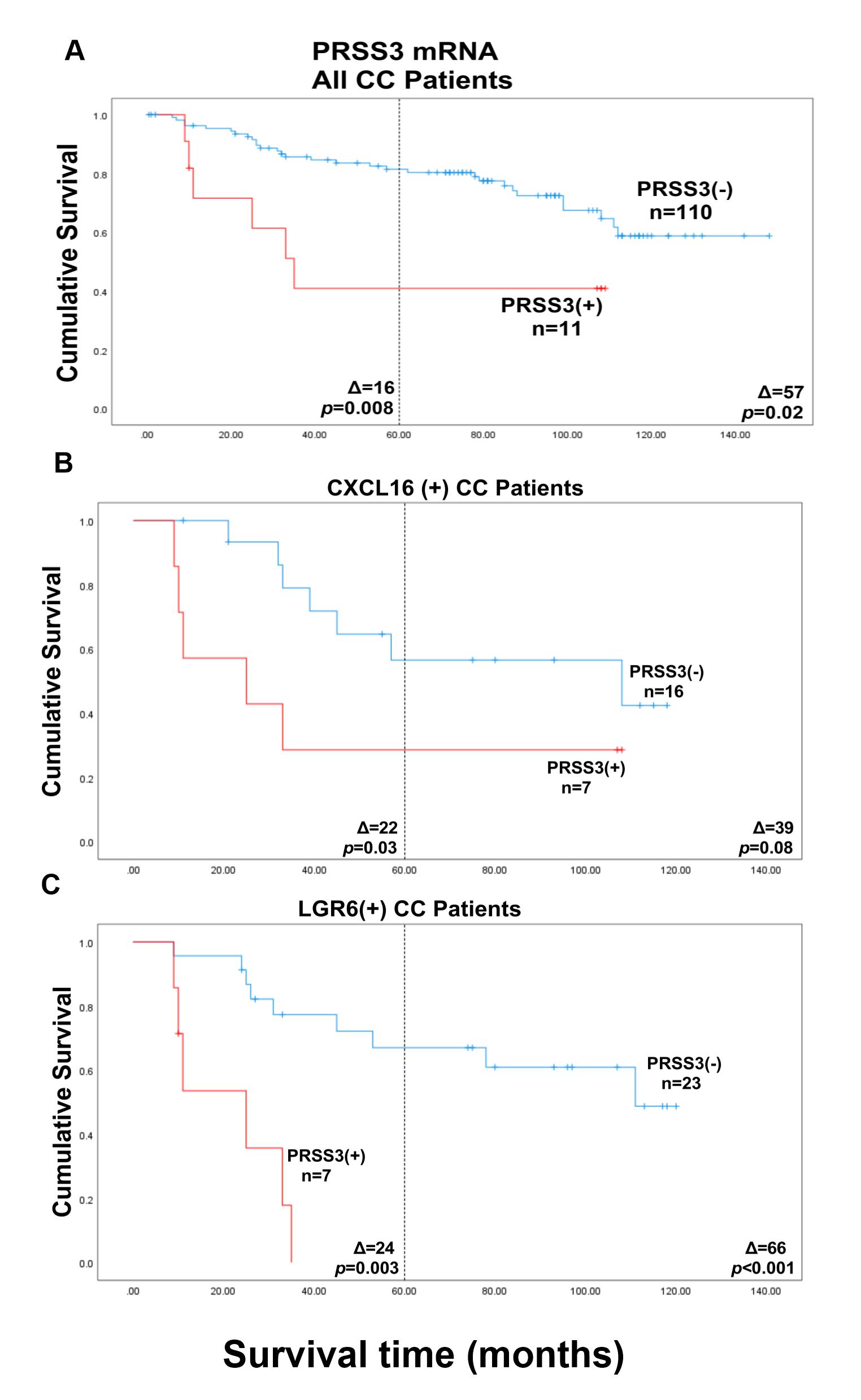

Supplement: Supplementary Figure 2 — Kaplan-Meier cumulative survival curves for CC patients divided into two groups, PRSS3(-) and PRSS3(+), according to the median value of PRSS3 mRNA expression in lymph nodes from CC patients in the CEA(+) group (2.3 mRNA copies/18S rRNA unit). (A), All 121 colon cancer patients in the study were represented by their highest lymph node. (B), patients with CXCL16 mRNA levels >11.4 mRNA copies/18S rRNA unit (CXCL16(+) CC patients; n = 23). (C), patients with LGR6 mRNA levels >0.0471 mRNA copies/18S rRNA unit (LGR6(+) CC patients; n = 30). The patients were followed for 12 years. The dashed line indicates a 5-year follow-up after surgery. Differences in disease-free survival time after surgery between the two groups are given as a Δ-value in months. p-values were calculated using log-rank test of survival analysis. n = number of patients in the respective group. [file Image2.jpeg]
