# Supplementary material for: Combined serine protease PRSS22 and CEA mRNA analysis identifies the majority of colon cancer patients that recur within 12 years
Source: Front Oncol. 2025 Aug 20;15:1628069. doi: 10.3389/fonc.2025.1628069 (PMC12406016; doi:10.3389/fonc.2025.1628069)
Supplement: Supplementary file 3 [file Table1.docx]

**Supplementary Table S1**. Comparative analysis of average survival time after surgery and risk for recurrence of disease of CC patients with PRSS3(-) and PRSS3(+) lymph nodes and in combination with CXCL16(+) and LGR6(+) subgroups.

| Patient Group | Category ^a^ | Number of Patients in Each Group Stratified by TNM stage | | | | Total | 5- year follow- up after surgery | | | | | 12- year follow- up after surgery | | | | |
| --- | --- | --- | --- | --- | --- | --- | --- | --- | --- | --- | --- | --- | --- | --- | --- | --- |
|  |  | Stage  I | Stage  II | Stage  III | Stage  IV |  | Disease- free survival ^b^ | | | Risk for recurrence ^c^ | | Disease- free survival ^b^ | | | Risk for recurrence ^c^ | |
|  |  |  |  |  |  |  | Average  (Months) | Difference  (Months) | *p*-value | Hazard Ratio (95%CI) | *p*-value | Average  (Months) | Difference  (Months) | *p*-value | Hazard Ratio (95%CI) | *p*-value |
| All CC Patients | PRSS3(-) | 22 | 51 | 32 | 5 | 110 | 53 | 16 | 0.008 | 3.1 | 0.01 | 113 | 57 | 0.02 | 2.7 | 0.03 |
|  | PRSS3(+) | 1 | 1 | 5 | 4 | 11 | 37 |  |  | (1.3-7.6) |  | 56 |  |  | (1.1-6.6) |  |
| CXCL16(+) CC patients ^d^ | PRSS3(-) | 2 | 7 | 5 | 2 | 16 | 51 | 22 | 0.03 | 3.5 | 0.04 | 82 | 39 | 0.08 | 2.7 | 0.09 |
|  | PRSS3(+) | 0 | 0 | 4 | 3 | 7 | 29 |  |  | (1.0-11.6) |  | 43 |  |  | (0.84-8.4) |  |
| LGR6(+) CC patients ^e^ | PRSS3(-) | 3 | 8 | 9 | 3 | 23 | 48 | 24 | 0.003 | 4.3 | 0.007 | 87 | 66 | <0.001 | 7.7 | <0.001 |
|  | PRSS3(+) | 0 | 0 | 3 | 4 | 7 | 24 |  |  | (1.5-12.6) |  | 21 |  |  | (2.3-26.0) |  |

^a^ CC patients divided into categories based on mRNA expression levels of PRSS3. PRSS3(-): the highest lymph node had <2.3 mRNA copies/18S rRNA unit; PRSS3(+): the highest lymph node had ≥2.3 mRNA copies/18S rRNA unit.

^b^ Mean survival time after surgery calculated by cumulative survival analysis according to Kaplan–Meier analysis.

^c^ Hazard ratio with 95% confidence interval (CI) for CC patients as calculated according to univariate COX regression analysis.

^d^ CC patient group with CXCL16 mRNA levels >11.4 mRNA copies/18S rRNA unit.

^e^ CC patient group with LGR6 mRNA levels >0.0471 mRNA copies/18S rRNA unit.
